# Pleiotropic function of the *SQUAMOSA PROMOTER-BINDING PROTEIN-LIKE* gene *TaSPL14* in wheat plant architecture

**DOI:** 10.1007/s00425-020-03531-x

**Published:** 2021-01-22

**Authors:** Jie Cao, Kaiye Liu, Wanjun Song, Jianing Zhang, Yingyin Yao, Mingming Xin, Zhaorong Hu, Huiru Peng, Zhongfu Ni, Qixin Sun, Jinkun Du

**Affiliations:** State Key Laboratory for Agrobiotechnology and Key Laboratory of Crop Heterosis and Utilization (MOE) and Beijing Key Laboratory of Crop Genetic Improvement, China Agricultural University, Beijing, 100193 People’s Republic of China

**Keywords:** Ethylene response genes, Panicle development, Plant height, TaSPL14, Thousand-grain weight, Tiller number, Wheat

## Abstract

**Main conclusion:**

The function of *SQUAMOSA PROMOTER-BINDING PROTEIN-BOX* gene *TaSPL14* in wheat is similar to that of *OsSPL14* in rice in regulating plant height, panicle length, spikelet number, and thousand-grain weight of wheat, but differs during tiller development. TaSPL14 may regulate spike development via ethylene-response gene *EIN3-LIKE 1* (*TaEIL1*), *ETHYLENE-RESPONSIVE TRANSCRIPTION FACTOR 2.11* (*TaRAP2.11*), and *ETHYLENE-RESPONSIVE TRANSCRIPTION FACTOR 1* (*TaERF1*), but not *DENSE AND ERECT PANICLE 1* (*TaDEP1*) in wheat.

**Abstract:**

The *SQUAMOSA PROMOTER-BINDING PROTEIN-LIKE* gene *OsSPL14* from rice is considered to be a major determinant of ideal plant architecture consisting of few unproductive tillers, more grains per spike, and high resistance of stems to lodging. However, the function of its orthologous gene, *TaSPL14*, in wheat is unknown. Here, we reported the functional similarities and differences between *TaSPL14* and *OsSPL14*. Similar to *OsSPL14* knock-outs in rice, wheat *TaSPL14* knock-out plants exhibited decreased plant height, panicle length, spikelet number, and thousand-grain weight. In contrast to *OsSPL14*, however, *TaSPL14* did not affect tiller number. Transcriptome analysis revealed that the expression of genes related to ethylene response was significantly decreased in young spikes of *TaSPL14* knock-out lines as compared with wild type. TaSPL14 directly binds to the promoters of the ethylene-response genes *TaEIL1*, *TaRAP2.11,* and *TaERF1,* and promotes their expression, suggesting that *TaSPL14* might regulate wheat spike development via the ethylene-response pathway. The elucidation of *TaSPL14* will contribute to understanding of the molecular mechanisms that underlie wheat plant architecture.

**Supplementary Information:**

The online version contains supplementary material available at 10.1007/s00425-020-03531-x.

## Introduction

The rice *SQUAMOSA PROMOTER-BINDING PROTEIN-LIKE* (*SPL*) gene *OsSPL14* (also known as *Ideal Plant Architecture 1*, *IPA1*) encodes a plant-specific transcription factor and is a new "Green Revolution" gene that plays a critical role in regulating rice ideal plant architecture (Wang and Wang 2017; Wang and Zhou 2018; Liu et al. 2019). Therefore, the optimally modulated expression of *OsSPL14* in rice could confer ideal plant architecture, including reduced tiller number, increased panicle primary branching, elevated lodging resistance of stems, and increased thousand-grain weight (Kerr and Beveridge 2017; Song et al. 2017; Zhang et al. 2017). Three natural alleles of *OsSPL14* have been identified from different high-yield rice lines. The *ipa1–1D* allele contains a nucleotide substitution (C to A) that affects miR156 targeting, leads to increased expression of *OsSPL14* in the shoot apex, and generates ideal plant architecture (Jiao et al. 2010; Wang et al. 2017). Reduced DNA methylation in the promoter region of the *WEALTHY FARMER’S PANICLE* (*WFP*) epigenetic allele shows an optimally increased expression of *OsSPL14* in young spikes, which strongly positively affects primary branch and grain number in rice (Miura et al. 2010). The *ipa1–2D* epigenetic allele exhibits an elevated expression of *OsSPL14* due to the presence of tandem repeat sequences in the promoter region, which leads to an open chromatin structure that controls tiller number, stem diameter, and panicle primary branch number in a dose-dependent manner (Zhang et al. 2017). In addition, base deletion in the exon of *OsSPL14* by gene editing causes a frameshift mutation, and the inactivation of OsSPL14 leads to a dwarf phenotype, with an extremely increased number of tillers, decreased plant height, panicle length, and spikelet number (Li et al. 2016). Collectively, these findings demonstrate that *OsSPL14* plays an important role in regulating ideal plant architecture in rice.

It was previously reported that OsSPL14 regulates ideal plant architecture by directly binding the GTAC core motif in the promoter sequences of target genes and controlling their expression (Lu et al. 2013). One such target gene, *DENSE AND ERECT PANICLE1* (*DEP1*), controls rice panicle size (Huang et al. 2009; Zhao et al. 2016). A higher expression of *OsSPL14* led to a significantly and slightly increased level of *DEP1* expression in shoot apices and young panicles, respectively (Lu et al. 2013). The panicle length of RIL-*IPA1*/*dep1* lines was significantly shorter than that in RIL-*IPA1*/*DEP1* lines, suggesting that OsSPL14 positively regulates *DEP1* in determining panicle length in rice (Huang et al. 2009; Lu et al. 2013). OsSPL14 regulates tiller number by directly activating rice *TEOSINTE BRANCHED 1* (*OsTB1*), which is a negative regulator of tillering in rice (Takeda et al. 2003; Lu et al. 2013). The expression of *OsTB1* was significantly higher in axillary buds of NIL-*ipa1* plants, which highly express *OsSPL14*, and the RIL-*ipa1*/*tb1* genotype could suppress the tiller phenotype of NIL-*ipa1* plants, indicating that the direct activation of *OsTB1* by OsSPL14 contributes to the low number of tillers in NIL-*ipa1* plants (Takeda et al. 2003; Minakuchi et al. 2010; Lu et al. 2013).

In wheat, only few genes have been reported to affect traits related to plant architecture, including *tiller inhibition 1* (*tin1*), *tiller inhibition 2* (*tin2*), *tiller inhibition 3* (*tin3*), and *fertile tiller inhibition* (*ftin*), which inhibit tiller formation (Peng et al. 1999; Spielmeyer and Richards 2004; Kuraparthy et al. 2007; Zhang et al. 2013), and the *Q* gene, which is associated with spike compactness (Feng et al. 2017). However, the effects of the *OsSPL14* ortholog *TaSPL14* on wheat plant architecture and yield remain largely unknown. The aim of this study was to analyze the function of *TaSPL14* in influencing wheat ideal plant architecture. We constructed *TaSPL14* knock-out (*taspl14*) lines using clustered regularly interspaced short palindromic repeats/CRISPR-associated protein 9 (CRISPR/Cas9), and found that *taspl14* plants had reduced plant height, panicle length, spikelet number, and thousand-grain weight compared with wild type, but a similar number of tillers. Comparison of the transcriptomes of young spikes of *taspl14* and wild-type plants indicated that genes involved in ethylene response were downregulated in *TaSPL14* knock-out lines, including *TaEIL1*, *TaERF1*, and *TaRAP2.11*. Furthermore, TaSPL14 directly activated the expression of these three genes in transactivation assays. These functions of TaSPL14 differ from those of OsSPL14. TaSPL14 regulates panicle length of wheat through regulating expression levels of genes associated with ethylene response, but not *TaDEP1*. Therefore, our data provide insights into the functions and mechanisms of TaSPL14 in the regulation of wheat plant architecture.

## Materials and methods

### Plant materials and growth conditions

*Triticum aestivum* L. (Fielder) and *TaSPL14* knock-out lines (*taspl14*) were grown in the experimental field of the China Agricultural University in Beijing (39°57′N, 116°17′E) for seed reproduction, and phenotypic and molecular analyses. Young spikes around 20–30 mm in length were harvested from wild-type and *taspl14* plants, and were immediately frozen in liquid nitrogen and stored at − 80 °C for subsequent experiments. For molecular analyses, Fielder and *taspl14* plants were grown in a controlled environment chamber in conditions of 45% humidity, 26/20 °C day/night temperature and 16 h light with 3000 lx illumination (Master GreenPower CG T 400 W E40, Philips).

### Phenotypic analysis

Plant height, panicle length, spikelet number, mean internode length (the ratio between panicle length and spikelet number), and thousand-grain weight were measured as the mean value for three independent T_2_ *TaSPL14* knock-out lines, #5-3, #12-4, and #13-4, during the harvest stage. Each independent line consisted of at least seven plants. Tiller number was measured at the filling stage, and was calculated as the mean value for 10 different plants from each knock-out line.

### Identification of *TaSPL* genes in wheat

To identify *SPL* gene family members in wheat, we downloaded the sequence of SQUAMOSA-PROMOTER-BINDING PROTEIN (SBP domain, PF03110) from Pfam (http://pfam.xfam.org/). All sequences that contained an SBP domain were obtained from the *Triticum aestivum* IWGSC database (http://plants.ensembl.org/biomart/) based on a hidden Markov model (HMM) profile with a cut-off *E* value of < 1 × 10^–5^ (Finn et al. 2011; Zhang et al. 2014; Clavijo et al. 2017). The presence of a conserved SBP domain in each predicted gene was confirmed using the Conserved Domain Database (CDD) website (http://www.ncbi.nih.gov/cdd). Analysis of homoeologous genes was performed using the Ensembl Plant website (http://plants.ensembl.org/index.html).

### Phylogenetic analysis of *SPL* genes

For phylogenetic analysis, the sequences of 19 *OsSPL* genes were obtained from GRAP (Xie et al. 2006). Phylogenetic analysis was performed using the amino-acid sequence of the encoded SBP domain (Li and Lu. 2014). We used the CDD website to confirm the encoded SBP domain amino-acid sequences of the identified SBP-box genes in rice and wheat. Multiple sequences’ alignment was performed with the ClustalW algorithm of MEGA5.02 (Li and Lu 2014). A phylogenetic tree was constructed using the neighbor-joining method and bootstrapping with 1000 replicates. The accession numbers of the *OsSPL* and *TaSPL* genes are shown in Suppl. Table S1.

### Reverse transcription quantitative real-time PCR (RT-qPCR) analysis

Total RNA was extracted from grains at 4, 15 and 25 days after pollination using the Quick RNA Isolation Kit (Beijing, China, Waryong, 0416-50) according to the manufacturer’s instructions, and Trizol (Invitrogen) reagent was used to isolate total RNA from young roots, stems at the jointing stage, and young leaves young spikes 5, 10, 20, and 30 mm in length. The RNA concentration was determined with a NanoDrop 2000 spectrophotometer (Thermo Scientific). First-strand cDNA was synthesized with an M-MLV Reverse Transcriptase kit (TaKaRa). RT-qPCR was performed using the CFX96 real-time system (Bio-Rad) in 10-μL reactions containing 2 μL each gene-specific primer, 1 μL cDNA template, 2 μL ddH_2_O, and 5 μL SYBR Premix EXTaq II (TaKaRa). The RT-qPCR conditions were 95 °C for 5 min, followed by 40 cycles of 95 °C for 30 s, 60 °C for 15 s, and 72 °C for 15 s. A melting curve was performed for each sample to confirm the specificity of the reactions. The relative expression level of each gene was calculated using the 2^−ΔCT^ method and was normalized to the CT value of the wheat *TaACTIN* gene (*TraesCS5B02G124100*). RT-qPCR was performed in triplicate for each sample (Zheng et al. 2019). Gene-specific primers are shown in Suppl. Table S2.

### Vector construction and wheat transformation

We used CRISPR/Cas9 to edit the genome sequence of *TaSPL14* (Xing et al. 2014), and the E-CRISP Design website (http://www.e-crisp.org/E-CRISP/designcrispr.html) to design specific single-guide RNAs (sgRNA) to the *TaSPL14*-coding sequence. We synthesized two reverse complementary sgRNA sequences with *Bsa*I cohesive ends, and ligated them to the intermediate vector pCBC-MT1T2 by PCR and subsequently inserted the fragments into the terminal vector pBUE411 as previously described (Xing et al. 2014). Plasmids were transformed into immature embryos of the wheat cultivar Fielder by *Agrobacterium*-mediated transformation using *Agrobacterium* strain EHA105. Phosphinothricin was used to screen for positive T_0_ transgenic plants (Ishida et al. 2015). DNA was isolated from T_0_ transgenic plants and wild-type Fielder using the CTAB method, and target fragments of *TaSPL14* were amplified using specific primers flanking the sgRNA. The *TaSPL14* amplicons were cloned into vectors and sequenced, and T_0_ transgenic plants were selected in which *TaSPL14-A*, *TaSPL14-B*, and *TaSPL14-D* were all mutated. We used the same method to identify T_1_ and T_2_ homozygous *TaSPL14* knock-out plants. The primers for vector construction and *TaSPL14* target fragments amplification are shown in Suppl. Table S2.

### RNA sequencing

We collected several young spikes (20–30 mm) from wild-type Fielder and one *TaSPL14* knock-out line (*taspl14-*#13-4) for RNA sequencing (RNA-seq). The extraction method of total RNA from young spikes using Trizol (Invitrogen) according to the manufacturer’s instructions, and each RNA sample was divided into two to give two technical replicates. At least 5 µg total RNA for each replicate was used to construct a cDNA library using Illumina Poly-A Purification TruSeq library reagents, on a NovaSeq platform. The RNA-seq data were analyzed as previously described (Quinlan and Hall 2010; Joshi and Fass 2011; Kim et al. 2013). The transcripts that were differentially expressed between *taspl14-*#13-4 and wild type were characterized with the Bioconductor package edgeR with an absolute value of log_2_ (fold change) ≥ 1 and a false discovery rate < 0.05 as cut-offs (Robinson et al. 2010). The gene ontology (GO) analysis was performed using agriGO v.2.0 with a cut-off of *P* < 0.05 (Yan et al. 2017).

### Electrophoretic mobility shift assay (EMSA)

The DNA-binding domain of TaSPL14 was expressed in BL21 *Escherichia coli* cells (Transgene, Beijing, China) by cloning the full-length open-reading frame of *TaSPL14-B* (*TraesCS5B02G265600*) into the *Bam*HI restriction enzyme site of pGEX6P-1 to generate a chimeric GST-TaSPL14 protein. Expression of recombinant proteins in Transetta (DE3) *E. coli* (Transgene) was induced with 0.1 mM isopropyl beta-D-thiogalactopyranoside (IPTG) in LB buffer overnight at 16 °C. Cells were harvested, washed, and suspended in 20 mL PBS buffer (137 mM NaCl, 2.7 mM KCl, 10 mM Na_2_HPO_4_, and 2 mM KH_2_PO_4_), and then were sonicated for 0.5–1 h in 30 mL PBS buffer containing 1 mM phenylmethylsulfonyl fluoride (PMSF), and 1/2 tablet of protease inhibitor cocktail (Roche). When the suspension became clear, cells were centrifuged at 13,000 g for 30–45 min at 4 °C, and the supernatant was collected and filtered into a 50-mL tube through a 0.22-mm membrane. The supernatant was incubated with 200-µL GST MAG Agarose Beads (Novagen) at 4 °C and shaken overnight. The GST beads were washed three times with 5 mL PBS buffer and then mixed with the supernatant. The mixture was incubated with 50 mM Tris–HCl (pH = 8.0) buffer containing 10 mM reduced glutathione at 4 °C for at least 7 h to elute TaSPL14-GST recombinant proteins. The NanoDrop 2000 spectrophotometer (Thermo Scientific) was used to determine the concentration of fusion proteins (Guo et al. 2015, 2018). The probes were labeled at the 5′ end with biotin, and unlabeled probes without biotin modification were also prepared that included competition probes with the same sequence as the labeled probes, and probes with a mutated core motif. All probes were synthesized by Invitrogen, and were made double-stranded by cooling from 100 °C to room temperature in annealing buffer. The LightShift^®^ Chemiluminescent EMSA Kit (Thermo Scientific) was used for EMSA; the DNA-binding system consisted of 1 × binding buffer (100 mM Tris, 500 mM KCl, 10 mM dithiothreitol at pH 7.5), 10% (v/v) glycerol, 0.5 mM EDTA, 10 mM ZnCl_2_ and 50 ng µL^−1^ poly dI-dC (Thermo Scientific), 2 µL fusion protein, 2 µL labeled probes (100 µM), and ddH_2_O to a final volume of 20 µL. Competition analysis was performed by adding fivefold and tenfold molar excesses of the unlabeled probes (50 µM) to the binding reaction 5 min before adding labeled probes. Reactions were incubated for 30 min at room temperature and were then electrophoresed on 6% native polyacrylamide gels. The gels were transferred to nylon membranes, and shifted complexes of labeled proteins and free probes were visualized using a chemiluminescence camera (Tanon, Beijing, China) (Guo et al. 2018). The sequences of the probes used for EMSA are listed in Suppl. Table S2.

### Transcriptional activity assays in *Nicotiana tabacum*

Dual-luciferase reporter assays were performed as described previously (Guan et al. 2014). The 2-kb promoter sequences of *TaEIL1* (*TraesCS2D02G099400*), *TaERF1* (*TraesCS2D02G414600*), and *TaRAP2.11* (*TraesCS2D02G202100*) were amplified from Fielder genomic DNA, and cloned into the *Bam*HI and *Pst*I sites of pGreenII containing the coding sequence of *LUC* from *Renilla reniformis* (REN) (Hellens et al. 2005) using the In-Fusion^®^ HD Cloning Kit (Clontech), to create the pGreenII reporters *pTaEIL1::LUC*, *pTaERF1::LUC* and *pTaRAP2.11::LUC* plasmids. pGreenII vector containing the coding sequence of *R. reniformis LUC* driven by the 35S promoter (Hellens et al. 2005) was used as a positive control. The full-length coding sequence of the *TaSPL14-B* sub-genome was amplified and cloned into the Supper1300 overexpression vector to generate the effector construct *35S::TaSPL14*. The following plasmids were introduced into *Agrobacterium* strain GV3101 (Hellens et al. 2005): Empty vector (Empty Supper1300) + *pTaEIL1::LUC*, *35S::TaSPL14* + *pTaEIL1::LUC*, empty vector (Empty Supper1300) + *pTaERF1::LUC*, *35S::TaSPL14* + *pTaERF1::LUC*, empty vector (Empty Supper1300) + *pTaRAP2.11::LUC*, *35S::TaSPL14* + *pTaRAP2.11::LUC*. *Agrobacterium* strains harboring these combinations of reporter plasmids and effector plasmids were co-infiltrated into young leaves of 4-week–old *Nicotiana benthamiana* plants. After infiltration for 48 h, infected leaves were harvested and the activity of *LUC* driven by the respective promoters of *TaEIL1*, *TaERF1,* or *TaRAP2.11* was quantified using the Dual-Luciferase Reporter Assay system (Promega) with the Synergy 2 Multi-Detection Microplate Reader (BioTek Instruments). Expression of *LUC* driven by the 35S promoter (*35S::REN*) was used as an internal control. Normalized data are presented as the ratio of the value of luciferase activity to that of the control *35S::LUC* signal from three independent biological samples. The primers for the constructs used for the transcriptional activity assays are shown in Suppl. Table S2.

## Results

### Identification of *TaSPL14* in wheat

**Fig. 1:**
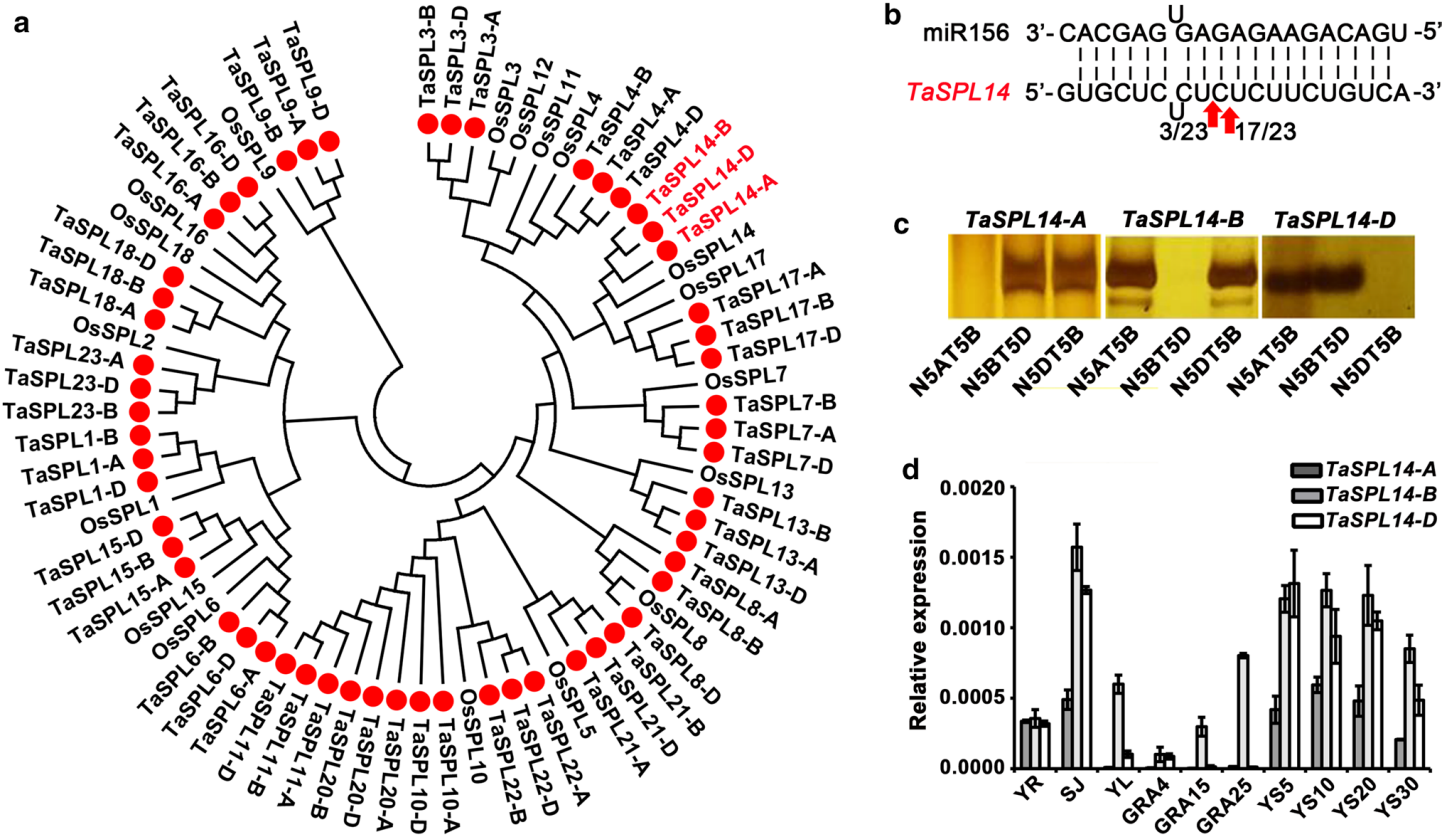
Identification and expression of *TaSPL14* in wheat. **a** Phylogenetic analysis of *SBP-box* genes in rice and wheat. The peptide sequences of the SBP domain of OsSPL and TaSPL proteins were aligned using ClustalW, the phylogenetic tree was constructed using MEGA5.02 with the neighbor-joining method, and the bootstrap test was performed with 1000 replicates. **b** miR156 and *TaSPL14* alignment and cleavage frequencies were detected using RLM-5′-RACE. The red arrows indicate the cleavage sites, and the numbers 3/23 and 17/23 show the ratios of the cleaved products from this site out of the total clones confirmed by sequencing. **c** The chromosomal locations of three homoeologous *TaSPL14* genes. The bands indicate amplification of homoeologous genes from the Chinese Spring (CS) null-tetrasomic lines. The nomenclature represents the different A, B, or D genomes: for example, N5AT5B represents nullisomic 5A-tetrasomic5B. **d** The expression of three homoeologous *TaSPL14* genes in different tissues: YR, young roots; SJ, stems at the jointing stage; YL, young leaves; GDAP4, GDAP15, and GDAP25 represent grains at 4, 15, and 25 days after pollination; YS5, YS10, YS20, and YS30 represent young spikes 5, 10, 20, or 30 mm in length. The data for each tissue consist of three biological replications and three technical replicates. The expression levels were normalized to that of wheat *TaACTIN*. Each bar in the graph corresponds to the mean value of three technical replicates ± SD

We annotated 56 sequences from the wheat genome (TGACv1 version) (Appels et al. 2018) that contained the SBP domain. Because common wheat (BBAADD) is a typical hexaploid species derived from three diploid ancestral species, *Triticum urartu* (AA), *Aegilops speltoides* (BB), and *Aegilops tauschii* (DD) (Petersen et al. 2006; Li et al. 2015; Ling et al. 2018; Zhang et al. 2018), the homoeologous genes among these 56 sequences from the three sub-genomes were grouped to 19 unique *TaSPL* genes, 10 of which had been previously isolated and named (Zhang et al. 2014) (Suppl. Table S1). We generated a phylogenetic tree based on the SBP domain of 19 OsSPL proteins from rice and 19 TaSPL proteins from wheat (Cardon et al. 1999; Xie et al. 2006; Yang et al. 2008). The remaining nine genes in the tree were named according to the orthologous genes of rice (Fig. 1a). We identified three homoeologous genes, *TraesCS5A02G265900*, *TraesCS5B02G265600*, and *TraesCS5D02G273900*, designated *TaSPL14-A*, *TaSPL14-B*, and *TaSPL14-D*, which showed high amino-acid similarity with *OsSPL14*. Previous studies suggested that *OsSPL14* was the target gene of OsmiR156 (Jiao et al. 2010). To verify whether miR156 could regulate *TaSPL14 *in vivo, we performed modified RNA ligase-mediated 5′-rapid amplification of cDNA ends (RLM-5′-RACE), and found that *TaSPL14* mRNA was cleaved by miR156 at the tenth nucleotide from the 5′ end (Fig. 1b).

We detected three genes homoeologous to *TaSPL14* located on chromosomes 5A, 5B, and 5D, respectively, using genomic DNA of a Chinese Spring (CS) line nullisomic–tetrasomic for chromosome group 5. When the corresponding chromosome was deleted from this CS line, no PCR amplicons could be generated (Fig. 1c). Next, we quantified the expression of *TaSPL14-A*, *TaSPL14-B*, and *TaSPL14-D* by RT-qPCR in different tissues, including young roots (YR), stems at the jointing stage (SJ), young leaves (YL), grains at 4, 15, and 25 days after pollination (GDAP), and young spikes 5 mm (YS5), 10 mm (YS10), 20 mm (YS20), or 30 mm (YS30) in length (Fig. 1d). The expression levels of *TaSPL14-B* and *TaSPL14-D* were higher than that of *TaSPL14-A* in the tissues examined. *TaSPL14-A* and *TaSPL14-D* were mostly expressed in stems and young spikes, whereas *TaSPL14-B* was abundant in stems, leaves, seeds, and young spikes, suggesting that these three homoeologous genes of *TaSPL14* contribute differently to wheat development.

### *TaSPL14* is similar to that of *OsSPL14* in regulating plant height, panicle length, spikelet number, and thousand-grain weight of wheat, but differs during tiller development

**Fig. 2:**
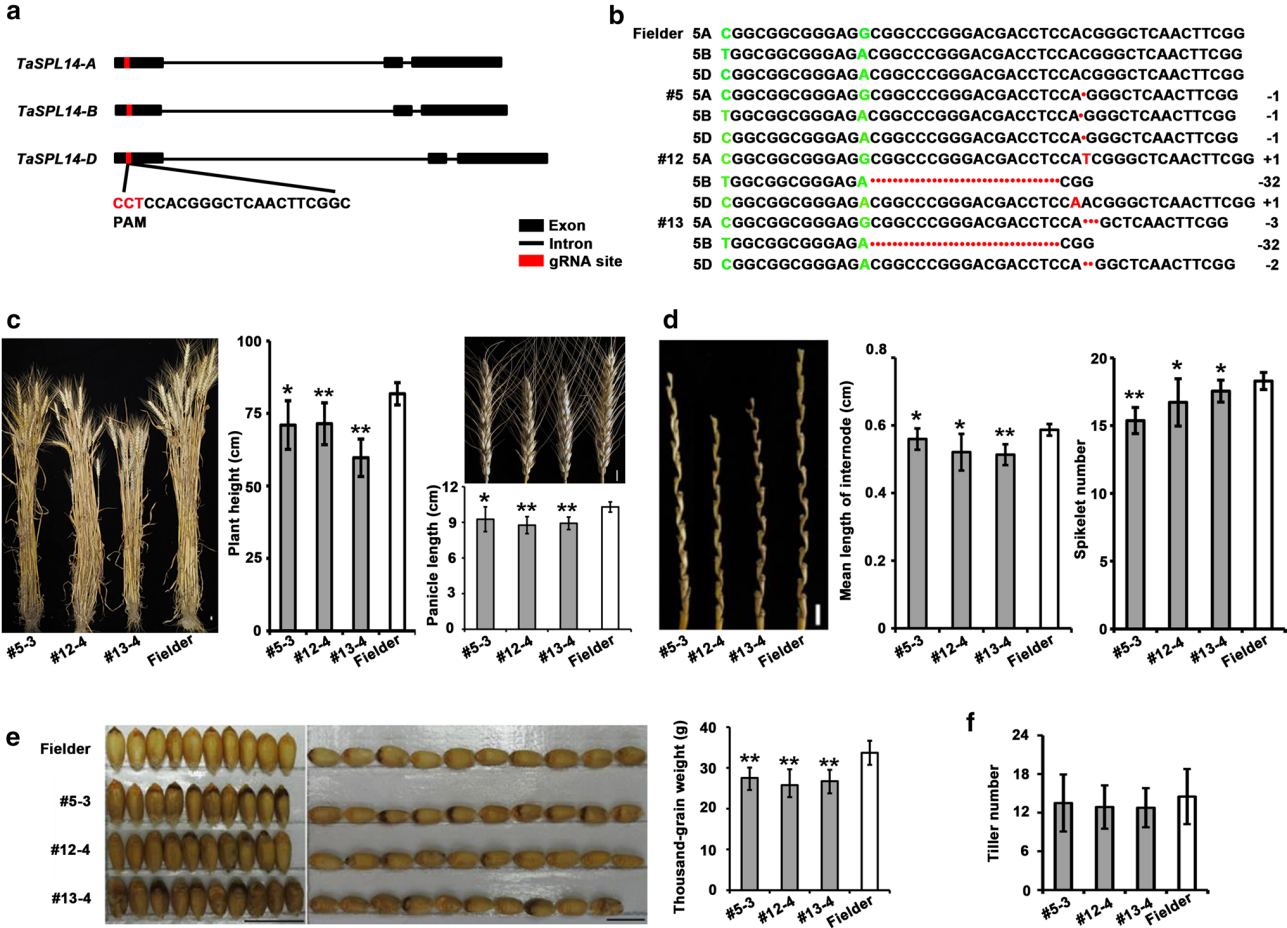
Phenotypic analysis of *TaSPL14* knock-out lines. **a** Schematic diagrams to illustrate sgRNA design for three *TaSPL14* homoeologs. Black boxes and lines represent exons and introns, respectively; red boxes denote the gRNA site. The protospacer-adjacent motif (PAM) sequence is highlighted in red letters, and the sgRNA sequence is in black letters. **b** The genotypes of three knock-out lines of *TaSPL14* were identified by sequencing. The numbers #5, #12, and #13 indicate three independent knock-out lines of *TaSPL14*; Fielder indicates wild type; " + " and " − " indicate insertions or deletions, respectively, caused by CRISPR/Cas9-induced mutations; numbers indicate the length of the insertion or deletion. **c** Plant height and panicle length of *TaSPL14* knock-out lines (#5–3, #12–4, and #13–4) and wild type (Fielder). Bar = 1 cm. **d** Mean internode length and spikelet number of *TaSPL14* knock-out lines (#5–3, #12–4, and #13–4) and wild type (Fielder). Bar = 1 cm. **e** Thousand-grain weight of *TaSPL14* knock-out lines (#5–3, #12–4, and #13–4) and wild type (Fielder). Bar = 1 cm. **f** Tiller number of *TaSPL14* knock-out lines (#5–3, #12–4, and #13–4) and wild type (Fielder). Bar = 10 cm. Each graph shows mean values ± SD for each sample (*n* ≥ 7). Single and double asterisks represent statistically significant differences determined by Student’s *t* test at *P* < 0.05 and *P* < 0.01, respectively

To investigate the biological function of *TaSPL14* in wheat, we generated *TaSPL14* knock-out plants using the CRISPR/Cas9 system (Xing et al. 2014). To simultaneously knock out the three homoeologous genes *TaSPL14-A*, *TaSPL14-B*, and *TaSPL14-D*, we designed a specific sgRNA that targets the conserved region within the first exon of the *TaSPL14*-*A*, *TaSPL14*-*B*, and *TaSPL14*-*D* sequences (Fig. 2a). The CRISPR/Cas9 vector *pBUE411::sgRNA* was transformed into wild-type Fielder by *Agrobacterium*-mediated transformation (Ishida et al. 2015). Three independent transgenic T_0_ plants were obtained and DNA sequencing for the *TaSPL14* loci revealed that all three homoeologous genes were simultaneously mutated, with frameshift mutations in the protein-coding sequence of *TaSPL14* knock-out lines #5 and #12, resulting in inactivated TaSPL14 proteins. The *TaSPL14* knock-out line #13 showed a 3-bp deletion in *TaSPL14-A* that did not cause a frameshift in the protein-coding region, whereas *TaSPL14-B* and *TaSPL14-D* each contained a frameshift mutation that produced an inactivated protein (Fig. 2b). Three independent T_2_ lines, #5-3, #12-4, and #13-4, were generated and sown in the field*.* All three *taspl14* lines showed significantly decreased plant height and panicle length compared with wide-type plants (Fig. 2c). Further observations revealed that the mean lengths of the rachis internode and the spikelet number were both significantly lower in *taspl14* than in the wild type (Fig. 2d). These results indicate that *TaSPL14* influences wheat spike development by affecting both rachis internode elongation and spikelet formation. Thousand-grain weight (TGW) in *TaSPL14* knock-out lines #5-3, #12-4, and #13-4 was markedly decreased, to 17.3, 22.88, and 23.7%, respectively, of that in wild type, indicating that *TaSPL14* influenced grain weight (Fig. 2e). Plant height, panicle length, and the number of flowers in rice *osspl14* (*ipa1*) lines were considerably lower than those in wild type (Li et al. 2016). Moreover, TGW increased in NIL OsSPL14^*ipa1*^, which has a higher expression level of *OsSPL14* (Jiao et al. 2010). Thus, our results indicate that *TaSPL14* possesses conserved functions with respect to those of *OsSPL14* in that it pleiotropically regulates plant height, panicle length, spikelet number, and TGW weight.

However, in contrast to *OsSPL14*, which regulates rice tillering (Song et al. 2017), *TaSPL14* is not associated with tiller development in wheat. The progeny of *tasp14* lines #5-3, #12-4, and #13-4 showed a similar tiller number to wild-type Fielder at the heading stage (Fig. 2f). These data indicate that the function of *TaSPL14* in tiller development differs from that of *OsSPL14*.

### TaSPL14 regulated ethylene-response genes, but not *TaDEP1* in wheat

**Fig. 3:**
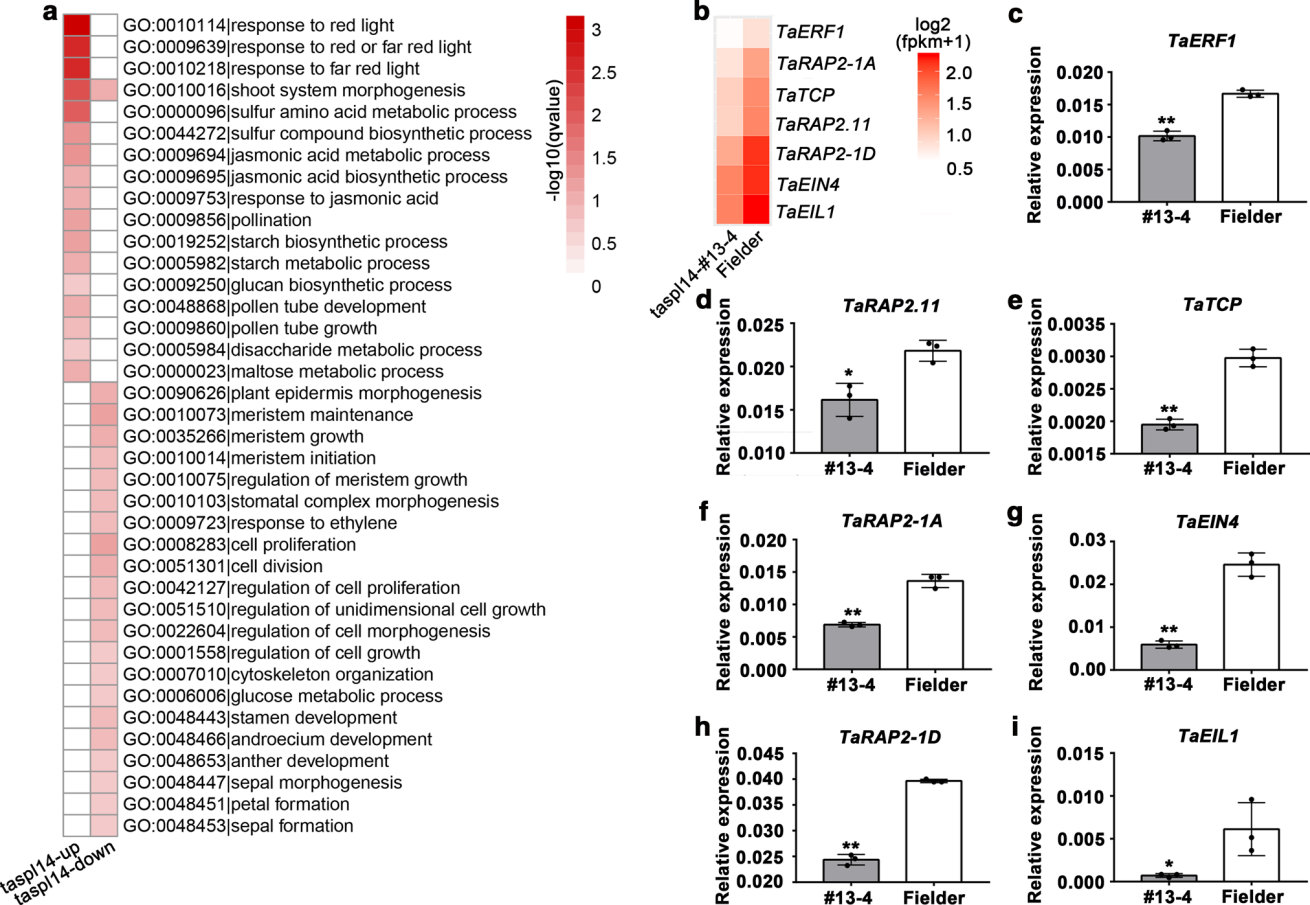
GO analysis and expression levels of differentially expressed ethylene-response genes in *taspl14-*#13–4 and wild-type Fielder. **a** GO analysis of ethylene-response genes that were differentially expressed in *taspl14-*#13–4 and wild type. The color in each cell indicates log_10_(*P* values) of the GO enrichment. **b** Heat map for the differentially expressed genes involved in ethylene response in the *taspl14-*#13–4 line and wild type. **C–i** Expression levels of differentially expressed genes involved in the ethylene response in young spikes of *taspl14-*#13–4 and wild type. The expression level was normalized to that of wheat *TaACTIN*. Each bar in the graph corresponds to the mean value ± SD of three technical replicates, denoted by dots. Single and double asterisks represent statistically significant differences determined by Student’s *t* test at *P* < 0.05 and *P* < 0.01, respectively

To understand how *TaSPL14* regulates spike development, we performed an RNA-seq experiment with young spikes (20–30 mm) from wild-type Fielder and *TaSPL14* knock-out line *taspl14-*#13-4. *TaSPL14-A* and *TaSPL14-B* were not differentially expressed in *taspl14-*#13-4 compared with wild type, and the expression of *TaSPL14-D* was lower in *taspl14-*#13-4 than in wild type (Suppl. Fig. S1). In total, 1,103 genes were downregulated and 228 genes were upregulated in *taspl14-*#13-4 compared with wild type (Suppl. Table S4). Further analysis of the Gene Ontology groups of differentially expressed genes showed that upregulated genes were mainly enriched in pathways related to responses to red or far-red light and jasmonic acid response, whereas the downregulated genes were mainly enriched in pathways related to meristem maintenance, meristem growth, cell proliferation, and ethylene response (Fig. 3a, Suppl. Table S3).

Among the downregulated genes, we observed that the expression of seven genes associated with ethylene response was statistically significantly lower in *taspl14-*#13-4 than in wild type (Fig. 3b): *ETHYLENE-RESPONSIVE TRANSCRIPTION FACTOR 1* (*TaERF1*, *TraesCS2D02G414600*), *ETHYLENE-RESPONSIVE TRANSCRIPTION FACTOR 2* (*TaRAP2.11*, *TraesCS2D02G202100*), *TEOSINTE BRANCHED1, CYCLOIDEA AND PCF TRANSCRIPTION FACTOR* (*TaTCP*, *TraesCS2D02G347200*), (*ETHYLENE-RESPONSIVE TRANSCRIPTION FACTOR 1* (*TaRAP2-1A*, *TraesCS1A02G231200*), *ETHYLENE INSENSITIVE 4* (*TaEIN4*, *TraesCS2D02G000500*), *ETHYLENE-RESPONSIVE TRANSCRIPTION FACTOR 1* (*TaRAP2-1D*, *TraesCS1D02G230900),* and *EIN3-LIKE 1* (*TaEIL1*, *TraesCS2D02G099400*). The downregulation of these genes in *taspl14-*#13-4 was validated by RT-qPCR analysis (Fig. 3c–i). Therefore, TaSPL14 regulates the expression of genes related to ethylene response.

**Fig. 4:**
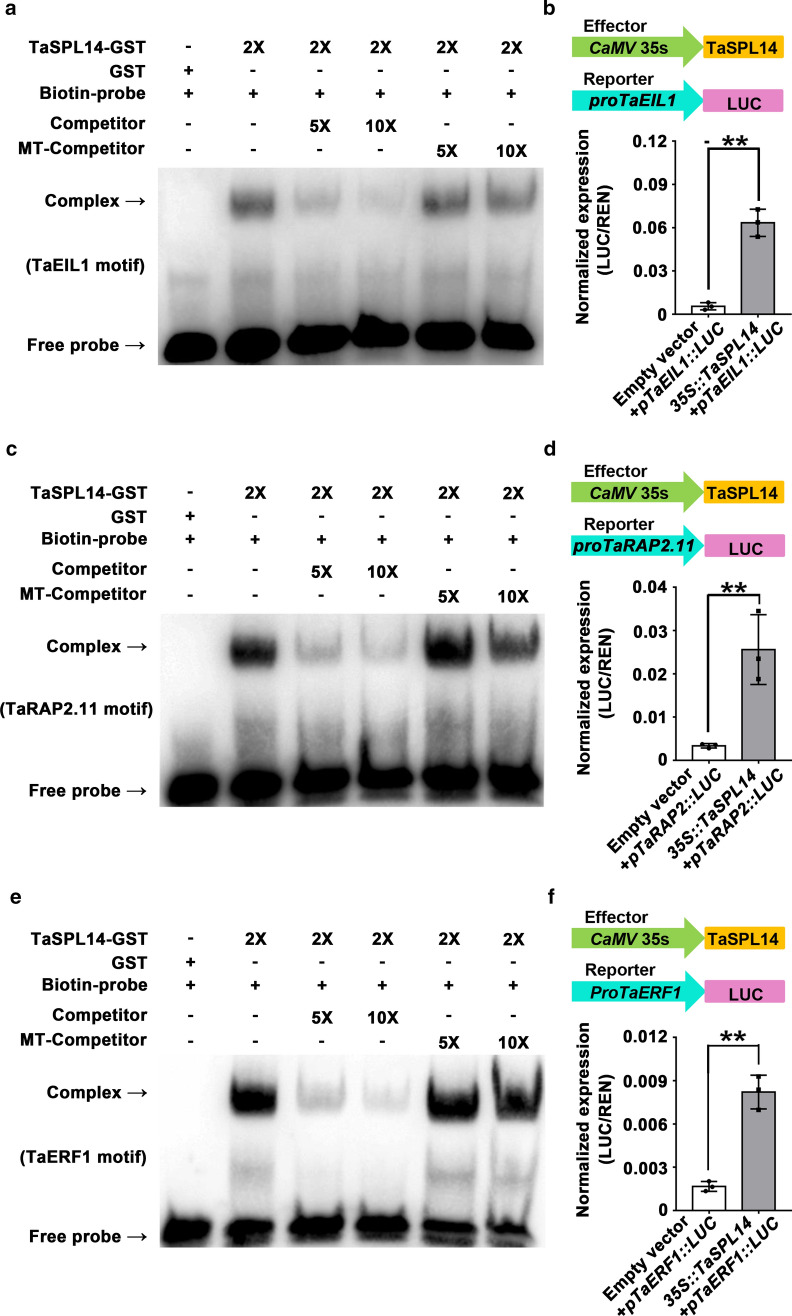
TaSPL14 directly binds and activates the promoters of *TaEIL1*, *TaRAP2.11*, and *TaERF1*. **a**, **c**, **e** Results of EMSAs using TaSPL14 recombinant proteins fused to GST and three biotin-labeled probes, competitor probes (biotin-unlabeled), and mutant competitor probes (biotin-unlabeled) derived from the *TaEIL1*, *TaERF1,* and *TaRAP2.11* promoters, to assess the binding of TaSPL14 to the GTAC core sequence in the promoter regions of *TaEIL1*, *TaRAP2.11*, and *TaERF1*. " + " and " − " indicate the presence and absence of corresponding recombinant proteins and probes; "2 × " indicates the amount of recombinant proteins in the reaction mixtures; "5 × " and "10 × " indicate fivefold and tenfold molar excesses of competitor or mutated competitor (MT) probes relative to the concentration of biotin-labeled probes. **b**, **d**, **f** TaSPL14 directly activated the expression of *TaEIL1*, *TaRAP2.11*, and *TaERF1*. Transactivation assays with *Nicotiana benthamiana* leaves infiltrated with the effector construct (*CaMV35s::TaSPL14*) combined with different reporter constructs (*proTaEIL1::LUC*, *proTaRAP2.11::LUC* or *proTaERF1::LUC*). Each bar in the graph corresponds to the mean value ± SD of three independent replicates, denoted by dots. The double asterisks represent significant differences determined by Student’s *t* test at *P* < 0.01

OsSPL14 can bind to the GTAC or TGGGCC/T core motif in the promoter regions of its target genes (Lu et al. 2013), which prompted us to analyze whether TaSPL14 regulates genes involved in ethylene responses by binding to their promoters. In the present study, the core GTAC motif was identified within the promoter sequences of *TaEIL1*, *TaRAP2.11*, and *TaERF1*, and we performed electrophoretic mobility shift assays (EMSAs) to determine whether TaSPL14 directly bound these sequences. Specific labeled and unlabeled probes were synthesized from the promoter regions of *TaEIL1*, *TaRAP2.11*, and *TaERF1*. Specific binding was observed between TaSPL14–GST fusion proteins and labeled probes for all three genes, and the EMSA band shift was prevented by the addition of an excessive molar concentration of unlabeled probes, but not mutated unlabeled probes, indicating that TaSPL14 bound the GTAC core motif in the promoters of *TaEIL1*, *TaRAP2.*11, and *TaERF1* (Fig. 4a–c). To confirm the regulation of *TaEIL1*, *TaRAP2.11*, and *TaERF1* by TaSPL14 in vivo, we performed transient transcriptional activity assays through *Agrobacterium*-mediated infiltration of *N. benthamiana* leaves. We constructed three plasmids that contained the *LUC* reporter gene driven by the promoter sequences of *TaEIL1* (*pTaEIL1::LUC*), *TaRAP2.11* (*pTaRAP2.11::LUC*), or *TaERF1* (*pTaERF1::LUC*) and an effector plasmid that overexpressed *TaSPL14* (*35S::TaSPL14*) (Fig. 4d–f). The results showed that co-expression of *35S::TaSPL14* with *pTaEIL1::LUC*, *pTaRAP2.11::LUC*, and *pTaERF1::LUC* led to a significant increase in *LUC* reporter gene activity compared with that of the empty-vector controls. This indicates that TaSPL14 can directly and dramatically elevate the expression of *TaEIL1*, *TaRAP2.11*, and *TaERF1* (Fig. 4d–f).

The *OsDEP1* gene is directly and positively regulated by OsSPL14, and it is an important regulatory gene that affects panicle architecture (Huang et al. 2009; Lu et al. 2013; Xu et al. 2016). To assess whether the SPL14–*DEP1* regulatory module is conserved between rice and wheat, we detected the expression of *TaDEP1* in *taspl14-*#13-4 and wild type. The three homoeologous genes *TaDEP1-A* (*TraesCS5A02G215100*), *TaDEP1-B* (*TraesCS5B02G208700*), and *TaDEP1-D* (*TraesCS5D02G216900*) are present in wheat (Vavilova et al. 2017; Dong et al. 2019), and RNA-seq demonstrated that they were not differentially expressed between young spikes of the *TaSPL14* knock-out line *taspl14-*#13-4 and the wild type, implying that TaSPL14 regulates spike development via other genes that are related to ethylene response, rather than via *TaDEP1* (Suppl. Fig. S2).

## Discussion

The *OsSPL14* gene is an essential component of the regulation of ideal rice plant architecture, including tiller development, panicle architecture, and the resistance of stems to lodging, and thereby substantially contributes to grain yield (Lu et al. 2013; Ordonio and Matsuoka 2017). However, the molecular and physiological functions of its ortholog, *TaSPL14*, in wheat are largely unknown. Therefore, in this study, we investigated the role and mechanisms of *TaSPL14* function in regulating wheat plant architecture.

**Fig. 5:**
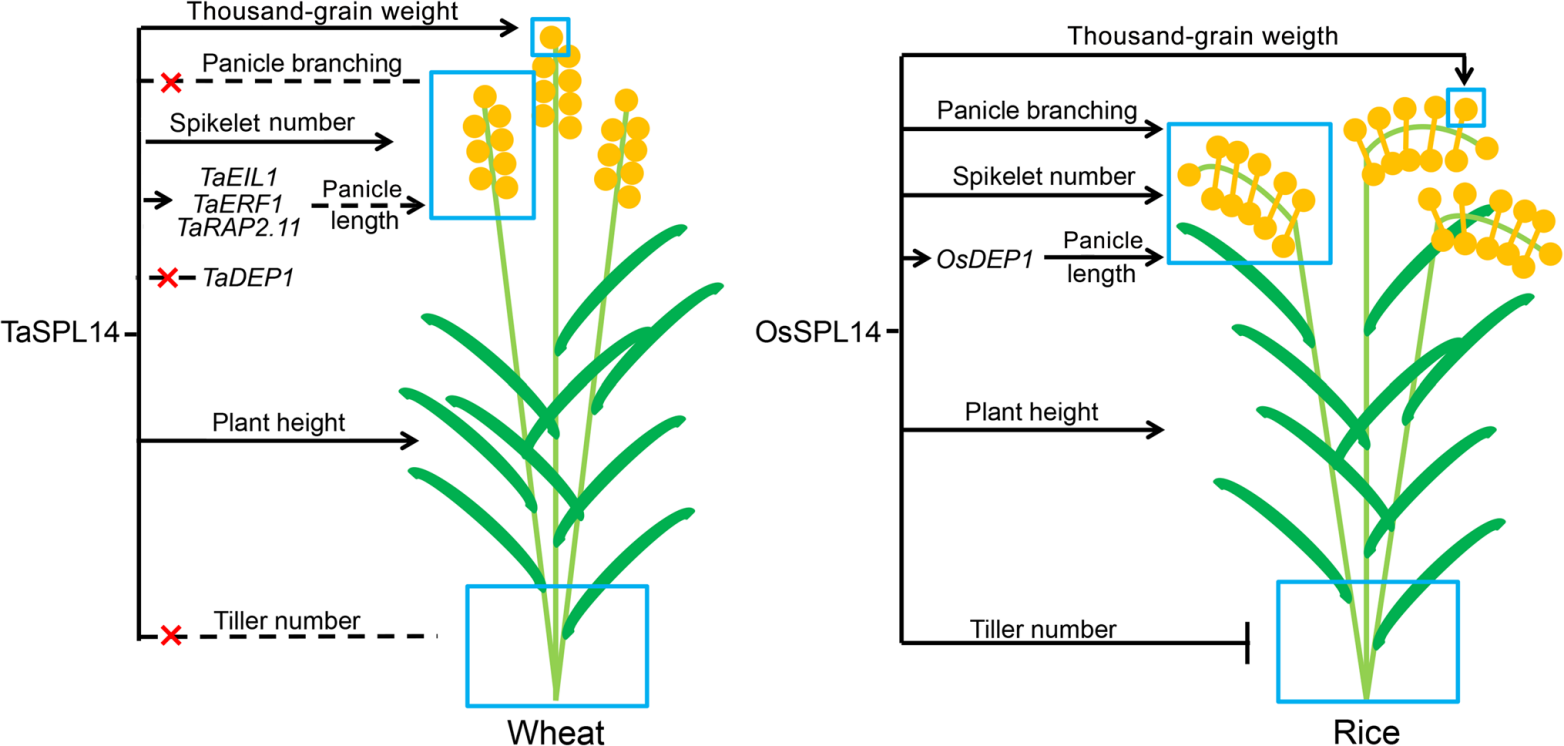
A summary of the functional similarities and differences between TaSPL14 and OsSPL14. Similar to OsSPL14 in rice, TaSPL14 acts as a regulator in wheat that pleiotropically regulates plant height, panicle length, spikelet number, and thousand-grain weight. Unlike OsSPL14 in rice, TaSPL14 in wheat had no effect on panicle branching and tiller number. OsSPL14 regulates panicle length by positively activating *OsDEP1*, but TaSPL14 might regulate wheat spike development via the ethylene-response-related genes *TaEIL1*, *TaERF1*, and *TaRAP2.11* and not via *TaDEP1*

Similar to OsSPL14, TaSPL14 acts as a major regulator of spike morphology. *taspl14* plants showed a reduction in panicle length and spikelet number, but no alterations in panicle branching (Fig. 2c, d). OsSPL14 influences rice panicle architecture not only by regulating panicle length and spikelet number, but also by strongly affecting primary branching (Jiao et al. 2010; Miura et al. 2010; Kim et al. 2018). Therefore, TaSPL14 and OsSPL14 show functional similarly in regulating panicle length and spikelet number per panicle. Notably, OsSPL14 is a positive regulator of *DEP1*, which is an important component of panicle architecture regulation (Lu et al. 2013). However, we observed that the expression level of *TaDEP1* was not affected in *taspl14* plants compared with that in wild type (Suppl. Fig. S2), suggesting that TaSPL14 might regulate spike development via pathways involving genes other than *DEP1*. We demonstrated that the expression levels of seven genes associated with ethylene response were significantly decreased in *taspl14-*#13-4 as compared to the wild type, and that TaSPL14 could directly and dramatically activate the promoter activities of *TaEIL1*, *TaERF1*, and *TaRAP2.11*. These data indicate that TaSPL14 might regulate spike development by regulating the ethylene-response pathway (Fig. 5).

In contrast to *OsSPL14* in rice, *TaSPL14* did not affect tillering in wheat. In rice, *osspl14* mutants showed two contrasting tiller-number phenotypes: *osspl14* mutants, carrying an amino-acid deletion in the OsSPL14 protein-coding sequence that maintained the activity of the protein, showed fewer tillers than wild type (Li et al. 2016); however, *osspl14* mutants, with a frameshift mutation that might completely inactivate the OsSPL14 protein, had a dwarf phenotype and more tillers than wild type (Li et al. 2016). Here, we observed that the tiller number of all *TaSPL14* knock-out lines was similar to that in wild type at the heading stage in the field (Fig. 2f), indicating a functional difference between *OsSPL14* and *TaSPL14*.

Three yield-positive *OsSPL14* alleles are known in rice *ipa1–1D*, the *WEALTHY FARMER’S PANICLE* (*WFP*) epigenetic allele and the *ipa1–2D* epigenetic allele all of which have been used in rice breeding (Jiao et al. 2010; Miura et al. 2010; Zhang et al. 2017). Introduction of the *OsSPL14*^ipa1^ allele into Xiushui11, a *japonica* rice variety, increased grain yield by ~ 10% in the field (Jiao et al. 2010). Moreover, introgression of the *OsSPL14*^WFP^ allele into different rice varieties significantly increased the number of primary branches and grains per plant (Ashikari et al. 2005; Ashikari and Matsuoka 2006; Miura et al. 2010; Kim et al. 2018). JYZK-3 and JYZK-4 are two hybrid varieties that carry *ipa1-2D* and show markedly improved yield performance in comparison with the control variety at different locations (Zhang et al. 2017). Thus, it is meaningful to identify natural variation related to the optimal expression levels of *TaSPL14* in young panicles, which could be used to design and generate high-yielding new varieties using marker-assisted selection.

### *Author contribution statement*

Z.N., Q.S., Y.Y., and D.J. designed the project and all experiments; J.C., K.L., W.S., and N.Z. carried out the experiments and analyzed experimental data; M.X., H.P., and Z.H. were responsible for wheat transformation; J.C., Y.Y., and D.J contributed to the writing and revision of the manuscript. All authors read and approved the content of this manuscript.

## Supplementary Information

Below is the link to the electronic supplementary material.

Supplementary file1 Suppl. Fig. S1 Expression of TaSPL14-A, TaSPL14-B and TaSPL14-D in young spikes of taspl14-#13-4. The horizontal axis represents three homoeologous genes of TaSPL14. The graphs represent the expression level in taspl14-#13-4 relative to that in wild-type Fielder. Each bar in the graph corresponds to the mean value ± SD for two technical replicates (TIF 456 KB)

Supplementary file2 Suppl. Fig. S2 The expression of TaDEP1-A, TaDEP1-B and TaDEP1-D in young spikes of taspl14-#13-4. The horizontal axis represents three homoeologous genes of TaDEP1. The graphs represent the expression level in taspl14-#13-4 relative to that in wild-type Fielder. Each bar in the graph corresponds to the mean value ± SD for two technical replicates (TIF 455 KB)

Supplementary file3 Suppl. Table S1 Accession numbers of SPL genes in rice and wheat. “*” indicates published TaSPL genes (Zhang et al. 2014) (XLSX 12 KB)

Supplementary file4 Suppl. Table S2 Gene primers used for 5'-RACE, RT-qPCR, vector construction and genomic DNA amplification (XLSX 13 KB)

Supplementary file5 Suppl. Table S3 GO analysis of differentially expressed genes (XLSX 14 KB)

Supplementary file6 Suppl. Table S4 Genes differentially expressed between taspl14-#13-4 and wild-type plants (XLSX 1337 KB)

## Data Availability

The datasets generated and analyzed during the current study are available in the NCBI (PRJNA629470) repository, https://www.ncbi.nlm.nih.gov/Traces/study/?acc=PRJNA629470.

## Abbreviations

IPA1: Ideal Plant Architecture 1
SBP: SQUAMOSA-BINDING PROTEIN
SPL: SQUAMOSA PROMOTER-BINDING PROTEIN-LIKE
