# Supplementary figures and images for: Pleiotropic function of the SQUAMOSA PROMOTER-BINDING PROTEIN-LIKE gene TaSPL14 in wheat plant architecture
Source: Planta. 2021 Jan 22;253(2):44. doi: 10.1007/s00425-020-03531-x (PMC7822796; doi:10.1007/s00425-020-03531-x)

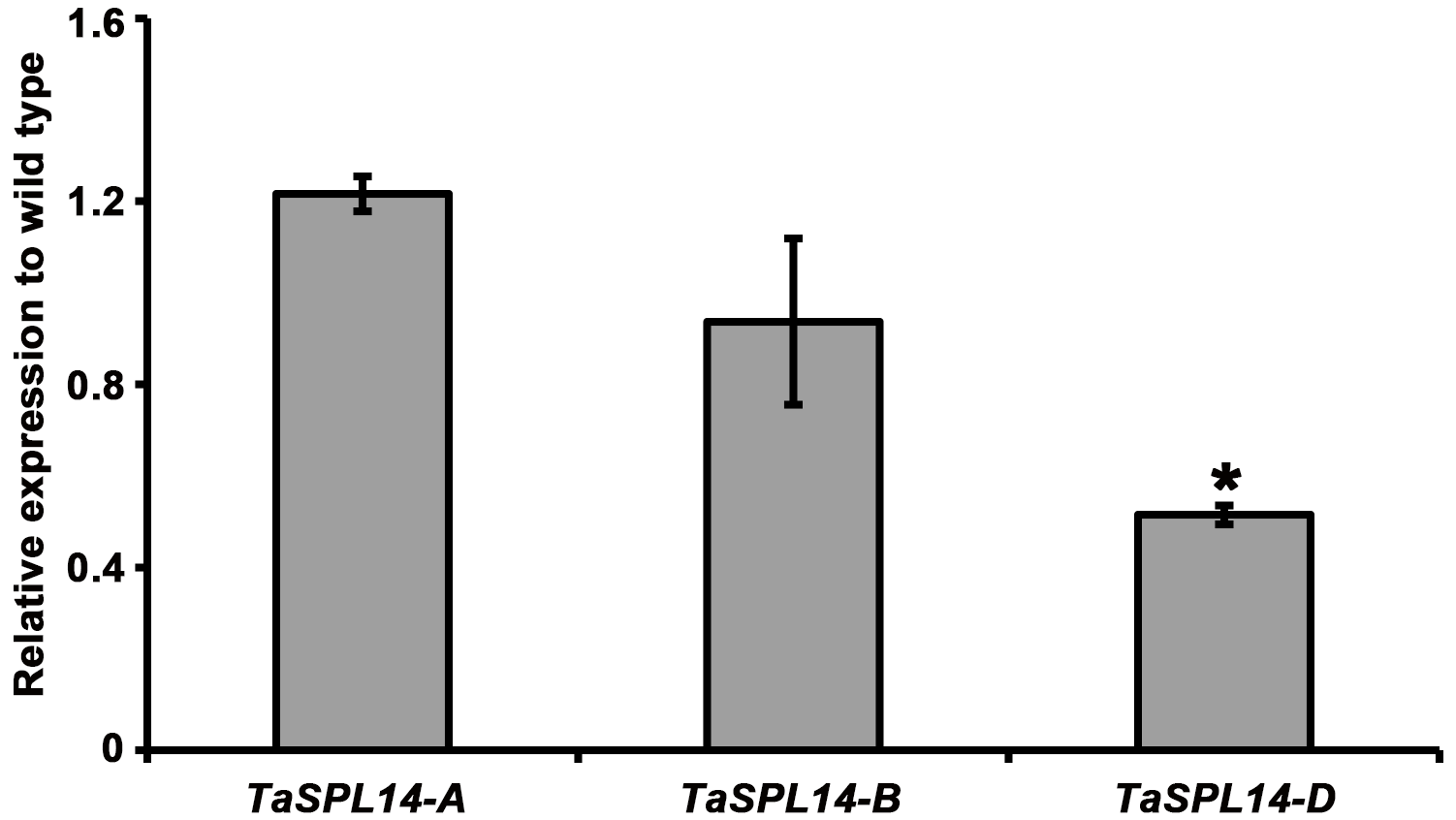

Supplement: Supplementary file 1 — Supplementary file1 Suppl. Fig. S1 Expression of TaSPL14-A, TaSPL14-B and TaSPL14-D in young spikes of taspl14-#13-4. The horizontal axis represents three homoeologous genes of TaSPL14. The graphs represent the expression level in taspl14-#13-4 relative to that in wild-type Fielder. Each bar in the graph corresponds to the mean value ± SD for two technical replicates (TIF 456 KB) [file 425_2020_3531_MOESM1_ESM.tif]

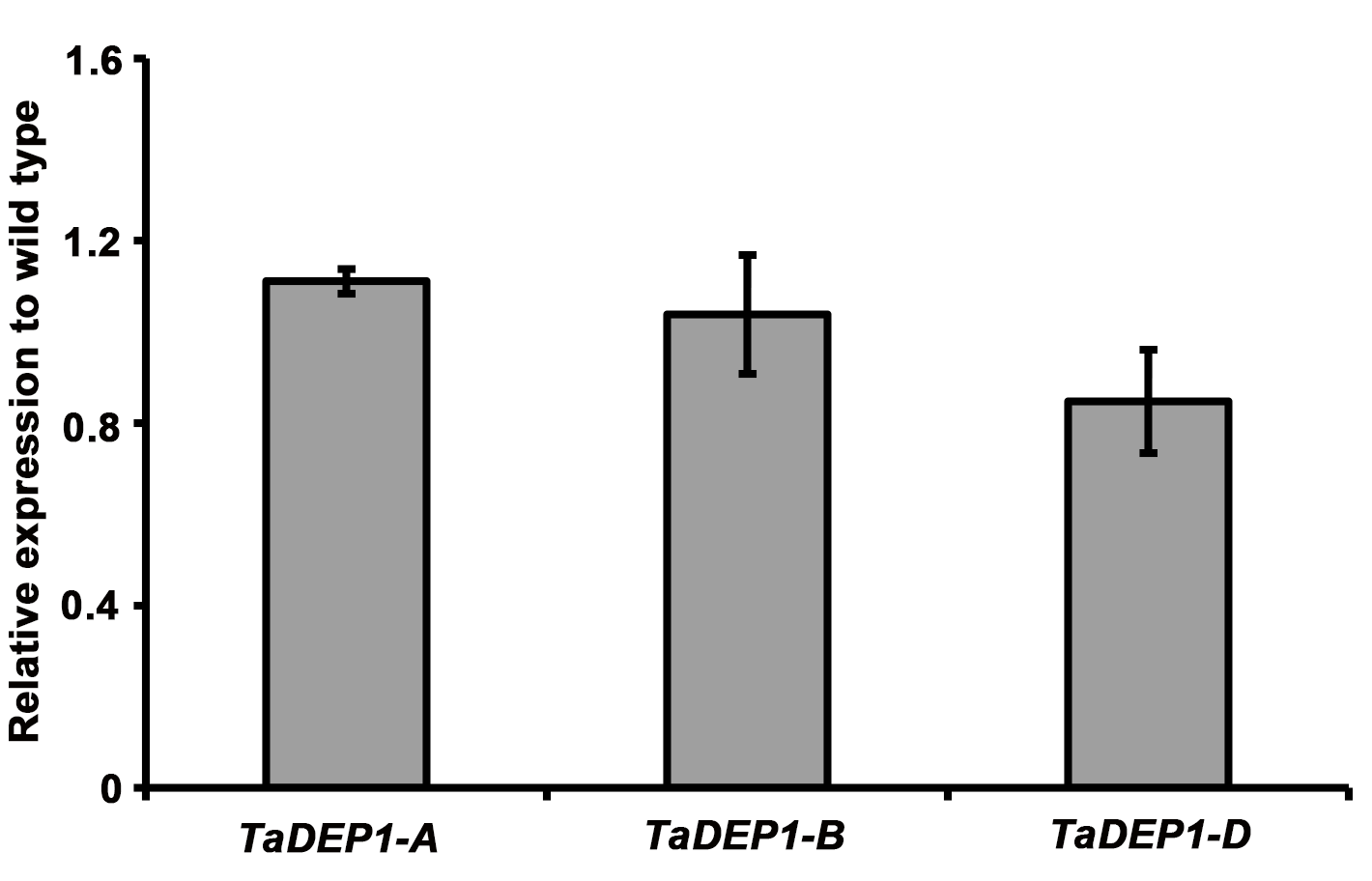

Supplement: Supplementary file 2 — Supplementary file2 Suppl. Fig. S2 The expression of TaDEP1-A, TaDEP1-B and TaDEP1-D in young spikes of taspl14-#13-4. The horizontal axis represents three homoeologous genes of TaDEP1. The graphs represent the expression level in taspl14-#13-4 relative to that in wild-type Fielder. Each bar in the graph corresponds to the mean value ± SD for two technical replicates (TIF 455 KB) [file 425_2020_3531_MOESM2_ESM.tif]
